# Crystal structure of bis­[*trans*-(1,4,8,11-tetra­aza­cyclo­tetra­decane-κ^4^ *N*)bis­(thio­cyanato-κ*N*)chromium(III)] tetra­chlorido­zincate from synchrotron data

**DOI:** 10.1107/S205698901500746X

**Published:** 2015-04-22

**Authors:** Dohyun Moon, Keon Sang Ryoo, Jong-Ha Choi

**Affiliations:** a Pohang Accelerator Laboratory, POSTECH, Pohang 790-784, Republic of Korea; b Department of Chemistry, Andong National University, Andong 760-749, Republic of Korea

**Keywords:** crystal structure, synchrotron radiation, cyclam, thio­cyanate ligand, *trans*-III configuration, chromium(III) complex, hydrogen bonding

## Abstract

The structure of the title compound, [Cr(NCS)_2_(cyclam)]_2_[ZnCl_4_] (cyclam = 1,4,8,11-tetra­aza­cyclo­tetra­decane, C_10_H_24_N_4_), has been determined from synchrotron data. The asymmetric unit contains two independent halves of the Cr^III^ complex cations and half of a tetra­chlorido­zincate anion. In each complex cation, the Cr^III^ atom is coordinated by the four N atoms of the cyclam ligand in the equatorial plane and by two N-bound NCS^−^ anions in a *trans* axial arrangement, displaying a distorted octa­hedral geometry with crystallographic inversion symmetry. The mean Cr—N(cyclam) and Cr—N(NCS) bond lengths are 2.065 (4) and 1.995 (6) Å, respectively. The macrocyclic cyclam moieties adopt centrosymmetric *trans*-III configurations with six- and five-membered chelate rings in chair and *gauche* configurations, respectively. The [ZnCl_4_]^2−^ anion, which lies about a twofold rotation axis, has a slightly distorted tetra­hedral geometry. The crystal packing is stabilized by hydrogen-bonding inter­actions between the N—H groups of the cyclam ligands, the S atoms of the NCS^−^ groups and the Cl^−^ ligands of the anion.

## Abstract 2

The Cr^III^ atoms in the title compound show a distorted octa­hedral coordination with four N atoms of the cyclam ligand in the equatorial plane and two N-coordinated NCS^−^ groups in axial positions. The macrocyclic ligands adopt *trans*-III configurations. The crystal packing is stabilized by N—H⋯S and N—H⋯Cl hydrogen bonds.

## Chemical context   

**Figure 1:**
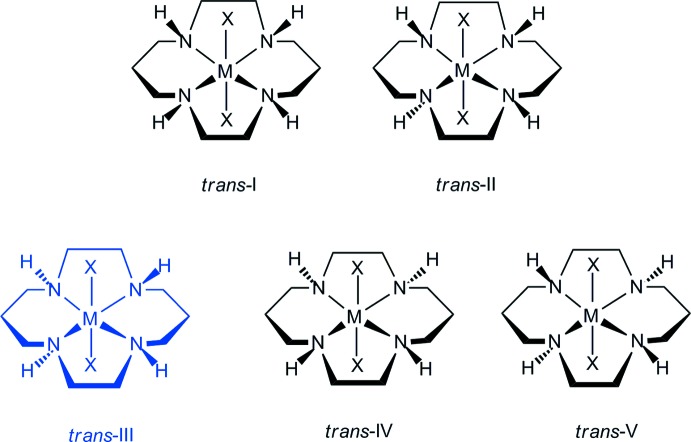
Possible configurations for *trans*-cyclam complexes with the *trans*-III configuration adopted by the title compound highlighted in blue.

In recent years, it has been found that cyclam (1,4,8,11-tetra­aza­cyclo­tetra­decane, C_10_H_24_N_4_) derivatives and their metal complexes exhibit anti-HIV activity (Ronconi & Sadler, 2007 ▸; De Clercq, 2010 ▸; Ross *et al.*, 2012 ▸). The cyclam derivatives inhibit the entry of the virus into white cells by binding to CXCR4, a chemokine receptor in the outer membrane. The strength of binding to the CXCR4 receptor correlates with the anti-HIV activity. The cyclam ligand has a moderately flexible structure, and can adopt both planar (*trans*) and folded (*cis*) configurations (Poon & Pun, 1980 ▸). There are five configurational *trans* isomers for this type of macrocycle, Fig. 1 ▸, that differ in the chirality of the *sec*-NH groups (Choi, 2009 ▸). The *trans*-V configuration can also fold to form the *cis*-V isomer (Subhan *et al.*, 2011 ▸). In addition, the thio­cyanate anion can be present in complexes as either a ligand or a non-coordinating anion (Moon *et al.*, 2013 ▸). Furthermore it can coordinate to metals as a terminal ligand through either the nitro­gen or the sulfur atoms, or can use both donor atoms and function as a bridging ligand.
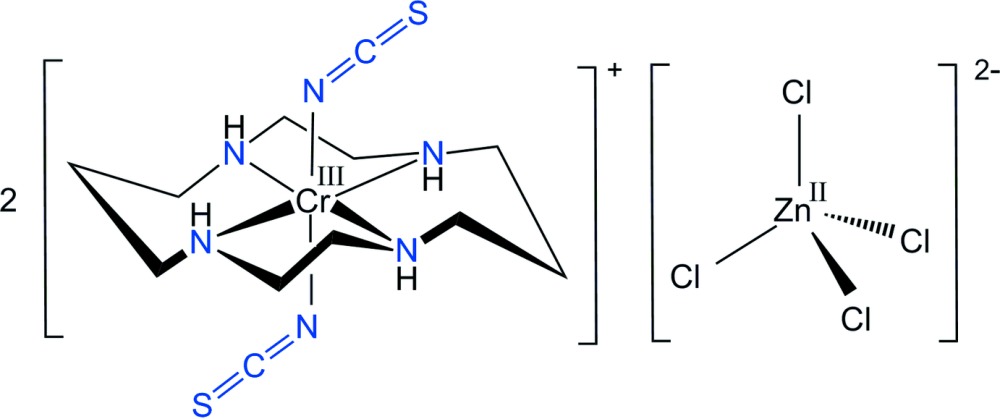


Counter-anionic species play a very important role in the coordination chemistry, pharmacy and biology (Fabbrizzi & Poggi, 2013 ▸) of metal complexes. Thus, we describe here the synthesis and structural characterization of *trans-*[Cr(NCS)_2_(cyclam)]_2_[ZnCl_4_], (I).

## Structural commentary   

**Figure 2:**
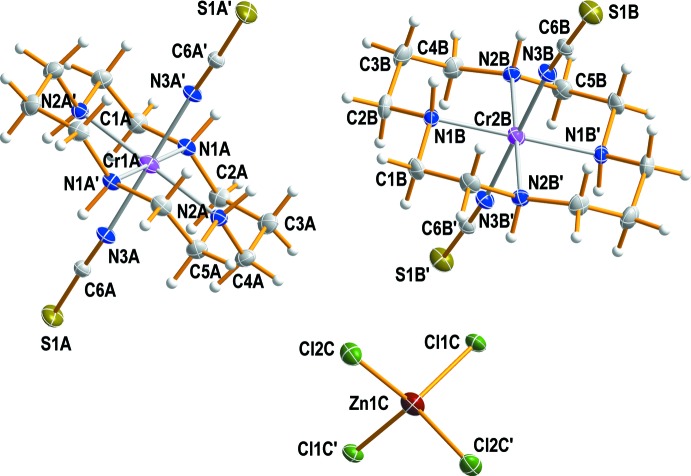
A perspective view (30% probability ellipsoids) of the two independent chromium(III) complex cations and the tetra­chlorido­zincate anion in (I). [Symmetry codes: (A′) *x* − 1, *y*, *z*; (B′) *x*, −*y*, *z* + 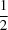; (C′) −*x* + 1, −*y* + 1, −*z* + 2.]

Each of the two *trans*-[Cr(NCS)_2_(cyclam)]^+^ cations in the structure of the title compound are generated by inversion symmetry, hence the configurations of the cyclam ligands can be described as *trans*-III, Fig. 1 ▸. The Cr^III^ cations, which are located on discrete inversion centres, are coordinated by the nitro­gen atoms of the cyclam ligands that occupy equatorial sites. Two thio­cyanate anions complete the distorted octa­hedral coordination sphere binding through their N atoms in a *trans* configuration. The single [ZnCl_4_]^2−^ anion, which lies about a twofold rotation axis, has slightly distorted tetra­hedral geometry and completes the complex salt. Fig. 2 ▸ shows an ellipsoid plot of (I), with the atom-numbering scheme. This is a second example of the structure of a *trans*-[Cr(NCS)]_2_(cyclam)]^+^ salt, but the previous example had a perchlorate counter-anion (Friesen *et al.*, 1997 ▸).

The Cr—N bond lengths from the donor atoms of the cyclam ligand range from 2.0614 (10) to 2.0700 (10) Å, and these lengths are comparable to those found in a range of related [Cr*L*_2_(cyclam)]^+^ complexes (Flores-Velez *et al.*, 1991 ▸; Friesen *et al.*, 1997 ▸; Choi, 2009 ▸; Choi, Oh, Suzuki *et al.*, 2004 ▸; Subhan *et al.*, 2011 ▸; Choi, Oh, Lim *et al.*, 2004 ▸). However, they are shorter than the bonds to a primary amine as found in the related complex *trans*-[CrCl_2_(Me_2_tn)_2_]_2_[ZnCl_4_] (Me_2_tn = [2,2-dimethylpropane-1,3-diamine]; Choi *et al.*, 2011 ▸). Furthermore, the mean Cr—N(NCS) distance of 1.9951 (11) Å is close the values found in other *trans/cis*-[Cr(NCS)_2_N_4_]^+^ cations (Moon & Choi, 2015 ▸; Choi & Lee, 2009 ▸; Moon *et al.*, 2013 ▸). As is normally found with cyclam complexes, the five-membered chelate rings adopt *gauche* configurations while the six-membered rings are in chair configurations. The average bite angles of the five- and six-membered chelate rings around the chromium(III) atoms are 85.51 (4) and 94.49 (4)°, respectively. The N-coordinated NCS ligands are almost linear, with N—C—S angles of 177.42 (12)° in cation *A* and 178.66 (12)° in cation *B*. The C6*A*—S1*A* bond length [1.6126 (12) Å] in the Cr1*A* complex cation is slightly longer than the C6*B–*-S1*B* bond length [1.6056 (12) Å] in the Cr2*B* complex cation. This elongation may be attributed to the weak hydrogen bond formed by S1*A* with the N2*A—*H2*A* group of the cyclam ligand.

## Supra­molecular features   

**Figure 3:**
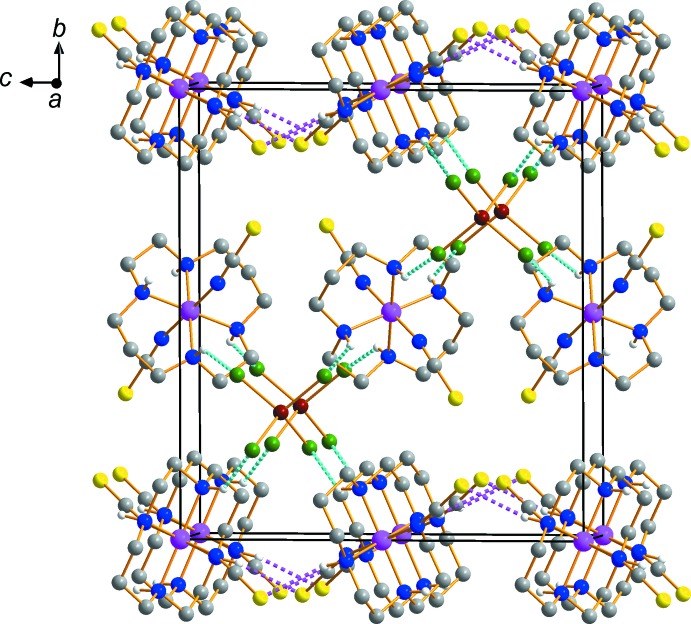
The mol­ecular packing in (I), viewed along the *a* axis. Dashed lines represent hydrogen-bonding inter­actions N—H⋯Cl (cyan) and N—H⋯S (purple), respectively. H atoms bound to C have been omitted.

Each complex mol­ecule forms three classical N—H⋯Cl hydrogen bonds between the amine groups of the cyclam ligand in each complex cation and the Cl atoms of the tetra­chlorido­zincate anion, Table 1 ▸ (Steed & Atwood, 2009 ▸). These hydrogen bonds link the cations and anions into a three-dimensional network as shown in Fig. 3 ▸ and help to stabilize the crystal structure.

## Database survey   

A search of the Cambridge Structural Database (Version 5.36, last update February 2015; Groom & Allen, 2014 ▸) gave only three hits for the [Cr(NCS)_2_(cyclam)]^+^ cation. Of these structures, *trans*-[Cr(NCS)_2_(cyclam)](ClO_4_) (Friesen *et al.*, 1997 ▸) adopts the *trans*-III configuration, similar to that adopted by the title compound, while *cis*-[Cr(NCS)_2_(cyclam)](ClO_4_) (Friesen *et al.*, 1997 ▸) and *cis*-[Cr(NCS)_2_(cyclam)](NCS) (Moon *et al.*, 2013 ▸), both adopt the folded *cis*-V configuration. No structure of a salt of [Cr(NCS)_2_(cyclam)]^+^ with the [ZnCl_4_]^2−^ anion was found.

## Synthesis and crystallization   

The free ligand cyclam was purchased from Strem Chemicals and used as provided. All chemicals were reagent-grade materials and were used without further purification. The starting material, *trans*-[Cr(NCS)_2_(cyclam)]ClO_4_, was prepared according to the literature (Friesen *et al.*, 1997 ▸). The perchlorate salt (0.33 g) was dissolved in 10 mL of 0.1 *M* HCl at 333 K and added to 7.5 mL of 6 *M* HCl containing 0.75 g of solid ZnCl_2_. The resulting solution was filtered, and allowed to stand at room temperature for two days to give pale-yellow crystals of (I) suitable for X-ray structural analysis.

## Refinement   

Crystal data, data collection and structure refinement details are summarized in Table 2 ▸. All H atoms were placed in geometrically idealized positions and constrained to ride on their parent atoms, with C—H = 0.97 Å and N—H = 0.98 Å, and with *U*_iso_(H) values of 1.2*U*_eq_ of the parent atoms.

## Supplementary Material

Crystal structure: contains datablock(s) I. DOI: 10.1107/S205698901500746X/sj5452sup1.cif

Structure factors: contains datablock(s) I. DOI: 10.1107/S205698901500746X/sj5452Isup2.hkl

CCDC reference: 1059896

Additional supporting information:  crystallographic information; 3D view; checkCIF report

## Figures and Tables

**Table 1:** Hydrogen-bond geometry (, )

*D*H*A*	*D*H	H*A*	*D* *A*	*D*H*A*
N1*A*H1*A*Cl2*C* ^i^	0.98	2.66	3.4510(12)	138
N2*A*H2*A*S1*A* ^ii^	0.98	2.60	3.4884(13)	151
N1*B*H1*B*Cl1*C* ^i^	0.98	2.58	3.4120(12)	143
N2*B*H2*B*Cl1*C* ^iii^	0.98	2.57	3.3944(13)	142

Symmetry codes: (i) 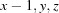; (ii) 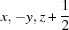; (iii) 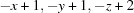.

**Table 2:** Experimental details

Crystal data	
Chemical formula	[Cr(NCS)_2_(C_10_H_24_N_4_)]_2_[ZnCl_4_]
*M*_r_	944.15
Crystal system, space group	Monoclinic, *P*2/*c*
Temperature (K)	260
*a*, *b*, *c* ()	7.9990(16), 16.532(3), 15.430(3)
()	101.36(3)
*V* (^3^)	2000.5(7)
*Z*	2
Radiation type	Synchrotron, = 0.610
(mm^1^)	1.07
Crystal size (mm)	0.22 0.19 0.12

Data collection	
Diffractometer	ADSC Q210 CCD area detector diffractometer
Absorption correction	Empirical (using intensity measurements) (*HKL3000sm* *SCALEPACK*; Otwinowski Minor, 1997 ▸)
*T*_min_, *T*_max_	0.801, 0.883
No. of measured, independent and observed [*I* > 2(*I*)] reflections	20982, 5738, 5500
*R*_int_	0.018
(sin /)_max_ (^1^)	0.706

Refinement	
*R*[*F* ^2^ > 2(*F* ^2^)], *wR*(*F* ^2^), *S*	0.025, 0.072, 1.06
No. of reflections	5738
No. of parameters	217
H-atom treatment	H-atom parameters constrained
_max_, _min_ (e ^3^)	0.60, 0.58

Computer programs: *PAL ADSC Quantum-210 ADX* (Arvai Nielsen, 1983 ▸), *HKL3000sm* (Otwinowski Minor, 1997 ▸), *SHELXT2014/5* (Sheldrick, 2015*a* ▸), *SHELXL2014/7* (Sheldrick, 2015*b* ▸), *DIAMOND* (Putz Brandenburg, 2014 ▸) nd *publCIF* (Westrip, 2010 ▸).

## supplementary crystallographic information

### Crystal data

**Table:**

[Cr(NCS)_2_(C_10_H_24_N_4_)]_2_[ZnCl_4_]	*F*(000) = 972
*M**_r_* = 944.15	*D*_x_ = 1.567 Mg m^−^^3^
Monoclinic, *P*2/*c*	Synchrotron radiation, λ = 0.610 Å
*a* = 7.9990 (16) Å	Cell parameters from 92486 reflections
*b* = 16.532 (3) Å	θ = 0.4–33.7°
*c* = 15.430 (3) Å	µ = 1.07 mm^−^^1^
β = 101.36 (3)°	*T* = 260 K
*V* = 2000.5 (7) Å^3^	Block, pale yellow
*Z* = 2	0.22 × 0.19 × 0.12 mm

### Data collection

**Table:**

ADSC Q210 CCD area-detector diffractometer	5500 reflections with *I* > 2σ(*I*)
Radiation source: PLSII 2D bending magnet	*R*_int_ = 0.018
ω scan	θ_max_ = 25.5°, θ_min_ = 2.4°
Absorption correction: empirical (using intensity measurements) (*HKL3000sm**SCALEPACK*; Otwinowski & Minor, 1997)	*h* = −11→11
*T*_min_ = 0.801, *T*_max_ = 0.883	*k* = −23→23
20982 measured reflections	*l* = −21→21
5738 independent reflections	

### Refinement

**Table:**

Refinement on *F*^2^	Hydrogen site location: inferred from neighbouring sites
Least-squares matrix: full	H-atom parameters constrained
*R*[*F*^2^ > 2σ(*F*^2^)] = 0.025	*w* = 1/[σ^2^(*F*_o_^2^) + (0.0404*P*)^2^ + 0.6258*P*] where *P* = (*F*_o_^2^ + 2*F*_c_^2^)/3
*wR*(*F*^2^) = 0.072	(Δ/σ)_max_ = 0.001
*S* = 1.06	Δρ_max_ = 0.60 e Å^−^^3^
5738 reflections	Δρ_min_ = −0.58 e Å^−^^3^
217 parameters	Extinction correction: *SHELXL2014/7* (Sheldrick 2015b), Fc^*^=kFc[1+0.001xFc^2^λ^3^/sin(2θ)]^-1/4^
0 restraints	Extinction coefficient: 0.013 (2)

### Special details

**Table:**

Geometry. All e.s.d.'s (except the e.s.d. in the dihedral angle between two l.s. planes) are estimated using the full covariance matrix. The cell e.s.d.'s are taken into account individually in the estimation of e.s.d.'s in distances, angles and torsion angles; correlations between e.s.d.'s in cell parameters are only used when they are defined by crystal symmetry. An approximate (isotropic) treatment of cell e.s.d.'s is used for estimating e.s.d.'s involving l.s. planes.

### Fractional atomic coordinates and isotropic or equivalent isotropic displacement parameters (Å^2^)

**Table:**

	*x*	*y*	*z*	*U*_iso_*/*U*_eq_	
Cr1A	0.0000	0.0000	0.5000	0.01897 (7)	
S1A	0.26852 (5)	0.12583 (3)	0.28897 (3)	0.04579 (10)	
N1A	0.02449 (13)	0.11498 (5)	0.55355 (7)	0.02520 (18)	
H1A	−0.0189	0.1129	0.6086	0.030*	
N2A	0.22186 (12)	−0.04305 (6)	0.57760 (7)	0.02639 (18)	
H2A	0.1956	−0.0559	0.6355	0.032*	
N3A	0.12441 (13)	0.03450 (6)	0.40583 (7)	0.0289 (2)	
C1A	−0.09496 (17)	0.16709 (7)	0.49075 (9)	0.0327 (2)	
H1A1	−0.0461	0.1798	0.4396	0.039*	
H1A2	−0.1155	0.2174	0.5193	0.039*	
C2A	0.20038 (17)	0.14817 (7)	0.57588 (9)	0.0340 (3)	
H2A1	0.1976	0.2001	0.6049	0.041*	
H2A2	0.2421	0.1571	0.5217	0.041*	
C3A	0.32286 (17)	0.09229 (9)	0.63584 (10)	0.0386 (3)	
H3A1	0.4276	0.1217	0.6577	0.046*	
H3A2	0.2733	0.0786	0.6865	0.046*	
C4A	0.36743 (16)	0.01431 (9)	0.59357 (10)	0.0359 (3)	
H4A1	0.4006	0.0267	0.5378	0.043*	
H4A2	0.4639	−0.0110	0.6319	0.043*	
C5A	0.26019 (17)	−0.12134 (7)	0.53750 (9)	0.0334 (2)	
H5A1	0.3391	−0.1528	0.5803	0.040*	
H5A2	0.3122	−0.1113	0.4868	0.040*	
C6A	0.18232 (14)	0.07189 (7)	0.35542 (8)	0.0263 (2)	
Cr2B	0.5000	0.5000	1.0000	0.01881 (7)	
S1B	0.26292 (6)	0.31238 (2)	1.17097 (3)	0.04295 (10)	
N1B	0.33254 (12)	0.45724 (6)	0.89068 (6)	0.02683 (18)	
H1B	0.2412	0.4284	0.9118	0.032*	
N2B	0.35373 (12)	0.60051 (6)	1.01461 (7)	0.02692 (19)	
H2B	0.2655	0.5822	1.0461	0.032*	
N3B	0.37356 (14)	0.43650 (7)	1.07593 (7)	0.0310 (2)	
C1B	0.43163 (18)	0.39638 (8)	0.85091 (8)	0.0351 (3)	
H1B1	0.5087	0.4235	0.8191	0.042*	
H1B2	0.3548	0.3627	0.8095	0.042*	
C2B	0.25141 (18)	0.51949 (9)	0.82674 (9)	0.0377 (3)	
H2B1	0.1736	0.4934	0.7787	0.045*	
H2B2	0.3385	0.5468	0.8020	0.045*	
C3B	0.15434 (18)	0.58129 (10)	0.87041 (10)	0.0429 (3)	
H3B1	0.0815	0.5525	0.9034	0.051*	
H3B2	0.0809	0.6119	0.8244	0.051*	
C4B	0.26361 (18)	0.64063 (8)	0.93259 (10)	0.0379 (3)	
H4B1	0.3467	0.6650	0.9026	0.045*	
H4B2	0.1917	0.6834	0.9479	0.045*	
C5B	0.46800 (18)	0.65534 (8)	1.07613 (9)	0.0353 (3)	
H5B1	0.4012	0.6952	1.1006	0.042*	
H5B2	0.5447	0.6835	1.0450	0.042*	
C6B	0.32530 (14)	0.38445 (7)	1.11521 (8)	0.0262 (2)	
Zn1C	1.0000	0.28259 (2)	0.7500	0.02829 (7)	
Cl1C	0.94451 (4)	0.36436 (2)	0.86092 (2)	0.03394 (8)	
Cl2C	0.77981 (5)	0.20141 (2)	0.68975 (3)	0.04336 (9)	

### Atomic displacement parameters (Å^2^)

**Table:**

	*U*^11^	*U*^22^	*U*^33^	*U*^12^	*U*^13^	*U*^23^
Cr1A	0.02262 (11)	0.01669 (11)	0.01964 (12)	−0.00101 (7)	0.00917 (8)	0.00082 (7)
S1A	0.0498 (2)	0.0544 (2)	0.03621 (19)	−0.01306 (16)	0.01597 (15)	0.01604 (15)
N1A	0.0311 (4)	0.0197 (4)	0.0267 (4)	−0.0016 (3)	0.0104 (4)	−0.0017 (3)
N2A	0.0272 (4)	0.0272 (4)	0.0255 (5)	0.0023 (3)	0.0069 (3)	−0.0002 (3)
N3A	0.0344 (5)	0.0289 (4)	0.0269 (5)	−0.0025 (4)	0.0142 (4)	0.0021 (4)
C1A	0.0423 (6)	0.0199 (5)	0.0370 (6)	0.0043 (4)	0.0105 (5)	0.0026 (4)
C2A	0.0372 (6)	0.0261 (5)	0.0404 (7)	−0.0106 (4)	0.0122 (5)	−0.0077 (5)
C3A	0.0330 (6)	0.0408 (7)	0.0402 (7)	−0.0073 (5)	0.0030 (5)	−0.0122 (5)
C4A	0.0248 (5)	0.0400 (6)	0.0426 (7)	−0.0012 (5)	0.0059 (5)	−0.0065 (5)
C5A	0.0346 (6)	0.0278 (5)	0.0381 (6)	0.0093 (4)	0.0079 (5)	−0.0004 (4)
C6A	0.0269 (5)	0.0287 (5)	0.0246 (5)	0.0001 (4)	0.0080 (4)	0.0015 (4)
Cr2B	0.02030 (11)	0.02070 (11)	0.01588 (12)	0.00103 (7)	0.00471 (8)	0.00120 (7)
S1B	0.0606 (2)	0.02721 (15)	0.0445 (2)	−0.01031 (14)	0.01873 (16)	0.00437 (13)
N1B	0.0273 (4)	0.0307 (5)	0.0210 (4)	−0.0015 (3)	0.0011 (3)	−0.0001 (3)
N2B	0.0268 (4)	0.0263 (4)	0.0282 (5)	0.0053 (3)	0.0067 (3)	−0.0006 (3)
N3B	0.0345 (5)	0.0326 (5)	0.0276 (5)	−0.0021 (4)	0.0106 (4)	0.0039 (4)
C1B	0.0422 (6)	0.0376 (6)	0.0252 (6)	−0.0019 (5)	0.0058 (5)	−0.0106 (5)
C2B	0.0404 (7)	0.0427 (7)	0.0243 (6)	0.0018 (5)	−0.0070 (5)	0.0040 (5)
C3B	0.0340 (6)	0.0483 (8)	0.0407 (7)	0.0109 (6)	−0.0067 (5)	0.0067 (6)
C4B	0.0388 (6)	0.0322 (6)	0.0404 (7)	0.0132 (5)	0.0024 (5)	0.0062 (5)
C5B	0.0401 (6)	0.0274 (5)	0.0390 (7)	0.0029 (5)	0.0087 (5)	−0.0086 (5)
C6B	0.0292 (5)	0.0258 (5)	0.0244 (5)	−0.0010 (4)	0.0073 (4)	−0.0035 (4)
Zn1C	0.03449 (11)	0.02582 (10)	0.02644 (11)	0.000	0.01061 (7)	0.000
Cl1C	0.03853 (15)	0.03419 (15)	0.03262 (15)	−0.00012 (11)	0.01562 (12)	−0.00580 (10)
Cl2C	0.05010 (19)	0.04102 (17)	0.04059 (19)	−0.01715 (14)	0.01290 (14)	−0.00567 (13)

### Geometric parameters (Å, º)

**Table:**

Cr1A—N3A^i^	1.9991 (11)	Cr2B—N1B^ii^	2.0614 (11)
Cr1A—N3A	1.9991 (11)	Cr2B—N1B	2.0614 (11)
Cr1A—N2A	2.0622 (11)	Cr2B—N2B^ii^	2.0700 (10)
Cr1A—N2A^i^	2.0623 (11)	Cr2B—N2B	2.0700 (10)
Cr1A—N1A^i^	2.0664 (10)	S1B—C6B	1.6056 (12)
Cr1A—N1A	2.0665 (10)	N1B—C2B	1.4836 (16)
S1A—C6A	1.6126 (12)	N1B—C1B	1.4861 (16)
N1A—C2A	1.4861 (16)	N1B—H1B	0.9800
N1A—C1A	1.4931 (16)	N2B—C4B	1.4838 (17)
N1A—H1A	0.9800	N2B—C5B	1.4887 (17)
N2A—C4A	1.4842 (16)	N2B—H2B	0.9800
N2A—C5A	1.4921 (15)	N3B—C6B	1.1614 (16)
N2A—H2A	0.9800	C1B—C5B^ii^	1.512 (2)
N3A—C6A	1.1591 (15)	C1B—H1B1	0.9700
C1A—C5A^i^	1.5106 (19)	C1B—H1B2	0.9700
C1A—H1A1	0.9700	C2B—C3B	1.519 (2)
C1A—H1A2	0.9700	C2B—H2B1	0.9700
C2A—C3A	1.521 (2)	C2B—H2B2	0.9700
C2A—H2A1	0.9700	C3B—C4B	1.522 (2)
C2A—H2A2	0.9700	C3B—H3B1	0.9700
C3A—C4A	1.5186 (19)	C3B—H3B2	0.9700
C3A—H3A1	0.9700	C4B—H4B1	0.9700
C3A—H3A2	0.9700	C4B—H4B2	0.9700
C4A—H4A1	0.9700	C5B—C1B^ii^	1.512 (2)
C4A—H4A2	0.9700	C5B—H5B1	0.9700
C5A—C1A^i^	1.5107 (19)	C5B—H5B2	0.9700
C5A—H5A1	0.9700	Zn1C—Cl2C^iii^	2.2632 (6)
C5A—H5A2	0.9700	Zn1C—Cl2C	2.2632 (6)
Cr2B—N3B^ii^	1.9911 (11)	Zn1C—Cl1C^iii^	2.2919 (5)
Cr2B—N3B	1.9911 (11)	Zn1C—Cl1C	2.2919 (5)
			
N3A^i^—Cr1A—N3A	180.0	N3B^ii^—Cr2B—N1B	91.31 (5)
N3A^i^—Cr1A—N2A	88.52 (4)	N3B—Cr2B—N1B	88.69 (5)
N3A—Cr1A—N2A	91.48 (4)	N1B^ii^—Cr2B—N1B	180.0
N3A^i^—Cr1A—N2A^i^	91.48 (5)	N3B^ii^—Cr2B—N2B^ii^	89.75 (5)
N3A—Cr1A—N2A^i^	88.52 (4)	N3B—Cr2B—N2B^ii^	90.25 (5)
N2A—Cr1A—N2A^i^	180.0	N1B^ii^—Cr2B—N2B^ii^	94.26 (4)
N3A^i^—Cr1A—N1A^i^	90.38 (4)	N1B—Cr2B—N2B^ii^	85.74 (4)
N3A—Cr1A—N1A^i^	89.62 (4)	N3B^ii^—Cr2B—N2B	90.25 (5)
N2A—Cr1A—N1A^i^	85.28 (4)	N3B—Cr2B—N2B	89.75 (5)
N2A^i^—Cr1A—N1A^i^	94.72 (4)	N1B^ii^—Cr2B—N2B	85.74 (4)
N3A^i^—Cr1A—N1A	89.62 (4)	N1B—Cr2B—N2B	94.26 (4)
N3A—Cr1A—N1A	90.38 (4)	N2B^ii^—Cr2B—N2B	180.0
N2A—Cr1A—N1A	94.72 (4)	C2B—N1B—C1B	113.21 (10)
N2A^i^—Cr1A—N1A	85.28 (4)	C2B—N1B—Cr2B	115.78 (8)
N1A^i^—Cr1A—N1A	180.0	C1B—N1B—Cr2B	104.87 (7)
C2A—N1A—C1A	113.14 (10)	C2B—N1B—H1B	107.5
C2A—N1A—Cr1A	116.31 (7)	C1B—N1B—H1B	107.5
C1A—N1A—Cr1A	105.85 (7)	Cr2B—N1B—H1B	107.5
C2A—N1A—H1A	107.0	C4B—N2B—C5B	113.94 (10)
C1A—N1A—H1A	107.0	C4B—N2B—Cr2B	117.13 (8)
Cr1A—N1A—H1A	107.0	C5B—N2B—Cr2B	105.60 (7)
C4A—N2A—C5A	113.86 (10)	C4B—N2B—H2B	106.5
C4A—N2A—Cr1A	115.67 (8)	C5B—N2B—H2B	106.5
C5A—N2A—Cr1A	106.39 (8)	Cr2B—N2B—H2B	106.5
C4A—N2A—H2A	106.8	C6B—N3B—Cr2B	163.19 (10)
C5A—N2A—H2A	106.8	N1B—C1B—C5B^ii^	108.88 (10)
Cr1A—N2A—H2A	106.8	N1B—C1B—H1B1	109.9
C6A—N3A—Cr1A	164.12 (10)	C5B^ii^—C1B—H1B1	109.9
N1A—C1A—C5A^i^	108.06 (10)	N1B—C1B—H1B2	109.9
N1A—C1A—H1A1	110.1	C5B^ii^—C1B—H1B2	109.9
C5A^i^—C1A—H1A1	110.1	H1B1—C1B—H1B2	108.3
N1A—C1A—H1A2	110.1	N1B—C2B—C3B	111.50 (11)
C5A^i^—C1A—H1A2	110.1	N1B—C2B—H2B1	109.3
H1A1—C1A—H1A2	108.4	C3B—C2B—H2B1	109.3
N1A—C2A—C3A	112.57 (10)	N1B—C2B—H2B2	109.3
N1A—C2A—H2A1	109.1	C3B—C2B—H2B2	109.3
C3A—C2A—H2A1	109.1	H2B1—C2B—H2B2	108.0
N1A—C2A—H2A2	109.1	C2B—C3B—C4B	115.66 (12)
C3A—C2A—H2A2	109.1	C2B—C3B—H3B1	108.4
H2A1—C2A—H2A2	107.8	C4B—C3B—H3B1	108.4
C4A—C3A—C2A	115.59 (12)	C2B—C3B—H3B2	108.4
C4A—C3A—H3A1	108.4	C4B—C3B—H3B2	108.4
C2A—C3A—H3A1	108.4	H3B1—C3B—H3B2	107.4
C4A—C3A—H3A2	108.4	N2B—C4B—C3B	111.82 (11)
C2A—C3A—H3A2	108.4	N2B—C4B—H4B1	109.3
H3A1—C3A—H3A2	107.4	C3B—C4B—H4B1	109.3
N2A—C4A—C3A	111.76 (10)	N2B—C4B—H4B2	109.3
N2A—C4A—H4A1	109.3	C3B—C4B—H4B2	109.3
C3A—C4A—H4A1	109.3	H4B1—C4B—H4B2	107.9
N2A—C4A—H4A2	109.3	N2B—C5B—C1B^ii^	107.44 (10)
C3A—C4A—H4A2	109.3	N2B—C5B—H5B1	110.2
H4A1—C4A—H4A2	107.9	C1B^ii^—C5B—H5B1	110.2
N2A—C5A—C1A^i^	108.33 (10)	N2B—C5B—H5B2	110.2
N2A—C5A—H5A1	110.0	C1B^ii^—C5B—H5B2	110.2
C1A^i^—C5A—H5A1	110.0	H5B1—C5B—H5B2	108.5
N2A—C5A—H5A2	110.0	N3B—C6B—S1B	178.66 (12)
C1A^i^—C5A—H5A2	110.0	Cl2C^iii^—Zn1C—Cl2C	107.27 (3)
H5A1—C5A—H5A2	108.4	Cl2C^iii^—Zn1C—Cl1C^iii^	114.02 (2)
N3A—C6A—S1A	177.42 (12)	Cl2C—Zn1C—Cl1C^iii^	107.01 (2)
N3B^ii^—Cr2B—N3B	180.00 (5)	Cl2C^iii^—Zn1C—Cl1C	107.01 (2)
N3B^ii^—Cr2B—N1B^ii^	88.69 (5)	Cl2C—Zn1C—Cl1C	114.02 (2)
N3B—Cr2B—N1B^ii^	91.31 (4)	Cl1C^iii^—Zn1C—Cl1C	107.71 (2)
			
C2A—N1A—C1A—C5A^i^	171.46 (10)	C2B—N1B—C1B—C5B^ii^	170.70 (11)
Cr1A—N1A—C1A—C5A^i^	42.96 (11)	Cr2B—N1B—C1B—C5B^ii^	43.59 (12)
C1A—N1A—C2A—C3A	−176.86 (10)	C1B—N1B—C2B—C3B	−178.93 (11)
Cr1A—N1A—C2A—C3A	−53.99 (13)	Cr2B—N1B—C2B—C3B	−57.79 (14)
N1A—C2A—C3A—C4A	69.83 (15)	N1B—C2B—C3B—C4B	72.48 (16)
C5A—N2A—C4A—C3A	−179.00 (11)	C5B—N2B—C4B—C3B	177.65 (11)
Cr1A—N2A—C4A—C3A	57.31 (14)	Cr2B—N2B—C4B—C3B	53.73 (14)
C2A—C3A—C4A—N2A	−71.67 (16)	C2B—C3B—C4B—N2B	−69.85 (17)
C4A—N2A—C5A—C1A^i^	−169.58 (11)	C4B—N2B—C5B—C1B^ii^	−172.02 (11)
Cr1A—N2A—C5A—C1A^i^	−40.99 (11)	Cr2B—N2B—C5B—C1B^ii^	−42.08 (11)

Symmetry codes: (i) −*x*, −*y*, −*z*+1; (ii) −*x*+1, −*y*+1, −*z*+2; (iii) −*x*+2, *y*, −*z*+3/2.

### Hydrogen-bond geometry (Å, º)

**Table:**

*D*—H···*A*	*D*—H	H···*A*	*D*···*A*	*D*—H···*A*
N1*A*—H1*A*···Cl2*C*^iv^	0.98	2.66	3.4510 (12)	138
N2*A*—H2*A*···S1*A*^v^	0.98	2.60	3.4884 (13)	151
N1*B*—H1*B*···Cl1*C*^iv^	0.98	2.58	3.4120 (12)	143
N2*B*—H2*B*···Cl1*C*^ii^	0.98	2.57	3.3944 (13)	142

Symmetry codes: (ii) −*x*+1, −*y*+1, −*z*+2; (iv) *x*−1, *y*, *z*; (v) *x*, −*y*, *z*+1/2.
